# Rectal Cancer Surgery in a District Hospital: Our Experience

**DOI:** 10.7759/cureus.75664

**Published:** 2024-12-13

**Authors:** Arslan Zahid, Atta ul Aleem Khalid, Khurram Waqas Anwer, Tasveer A Javed, Muzaffar Ahmad

**Affiliations:** 1 Surgery Department, Northern Lincolnshire and Goole NHS Trust, Scunthorpe, GBR

**Keywords:** abdomino-perineal resection, anterior resection, colorectal cancer, district hospital, hartmann procedure, rectal cancer

## Abstract

Introduction

Rectal cancer forms a significant proportion of newly diagnosed colorectal cancer. Treatment of rectal cancer is multi-modal, but surgery remains the cornerstone of treatment of rectal cancer and has undergone significant changes in the last three decades. The advent of minimally invasive techniques has revolutionised the landscape of surgery of the rectum. There is now a growing push to centralise rectal cancer surgery to tertiary centres only. We present the results of rectal cancer surgery from our district hospital.

Methods

This is a single-centre retrospective review of patients undergoing rectal cancer surgery from January 2018 to December 2019 at Northern Lincolnshire and Goole Trust.

Results

A total of 104 patients were included, with a mean age of 69.13 years (median 70, range 45-98 years). Of the patients, 65 (62.5%) patients were male and 39 (37.5%) patients were female. Neoadjuvant therapy was given to 34% of patients, while 66% of patients underwent surgery first. Anterior resection was performed in 64% of patients, abdomino-perineal resection was performed in 24% of patients, and Hartmann’s type operations were performed in 9% of patients. Median length of stay was 9 days (range 2-78 days). Morbidity was 24%, and five patients had anastomotic leaks, of whom three had radiological drain insertion and two required re-operation. Mortality was 2.,8% and re-operations were performed in 2% of patients.

Conclusion

Rectal cancer surgery can be safely undertaken in district hospitals with adequately trained surgeons using a multi-disciplinary approach.

## Introduction

Colorectal cancer is the eighth most common cancer and the 10th leading cause of cancer-related deaths in the world [1]. In the United Kingdom, it is the fourth most common cancer and constitutes 11% of all newly diagnosed cancers. Around 44,100 new cases of colorectal cancer are diagnosed each year [2]. Cancer of the rectum forms a significant proportion of new bowel cancers. Rectal surgery remains one of the main areas of interest in colorectal surgery and arguably has seen the most advancement in terms of new operative techniques in the last three decades. Minimal access surgery has completely changed the landscape of rectal cancer surgery, adding more techniques to the arsenal of modern colorectal surgeons [3,4]. Laparoscopic rectal surgery in particular has been widely adopted due to various benefits including quicker recovery and better visualisation in the narrow confines of the pelvis [5]. More procedures are now being performed using robotic and transanal approaches than ever before. However, access to these new platforms is still not as widespread as laparoscopy.

The advent of robotics in particular has had a profound effect on surgery of the rectum. Advantages of using the robot in rectal surgery include more dynamic movements in the pelvic space, better visualisation, and more ergonomic operating for the surgeon. Whether this translates into better outcomes is still being debated [6]. The cost of the robotic platforms is also one of the factors which has limited uptake as compared to laparoscopy which has significantly lower costs. Currently, robots are not available in every hospital, but most tertiary centres have access to robotic surgery. Using a robot for rectal surgery might become the norm in the future, but, currently, laparoscopic and open approaches are still being used in majority of hospitals where robotic facilities are not available [7].

In this era of hyper-specialisation, there has been a push towards centralisation of rectal cancer surgery [8,9]. Allowing only tertiary centres to operate on rectal cancer has some merit as tertiary care centres have more resources as compared to district general hospitals, resulting in high volumes; however, some other factors should also be considered [10]. Access to these tertiary care centres might also be limited for patients in rural areas, which might influence the decision-making process of the patients [11]. The de-skilling of the colorectal workforce may also be a consequence, which will impact quality of care in district hospitals in the future where emergency care is provided. District hospitals are not as rich in resource as tertiary centres but they continue to provide good quality care to rectal cancer patients [12]. We believe that rectal cancer can still be operated on in district general hospitals by experienced and competent surgeons and good basic facilities including imaging modalities, intensive care unit, and access to interventional radiology and oncological facilities.

We present our experience from Northern Lincolnshire and Goole, where we operate across two sites with a total bed capacity of 800 beds and 8 operating theatres. We serve a population of approximately half a million people. We are equipped with modern computed tomography and magnetic resonance imaging scanners. Our multi-disciplinary team (MDT) comprises of colorectal surgeons, radiologists, oncologists, and cancer nurse specialists. The objective of this study is to evaluate the outcomes of rectal cancer surgeries performed at Northern Lincolnshire and Goole Trust, focusing on patient demographics, surgical techniques, postoperative complications, and overall safety in a district hospital setting. We present our results from 2018 to 2019.

## Materials and methods

This was a single-centre retrospective review of the patients undergoing rectal cancer surgery from January 2018 to December 2019 at the Northern Lincolnshire and Goole Trust. All patients were discussed in the Colorectal Cancer Multidisciplinary Tumor board meeting, and treatment plans were formulated at the meeting in keeping with NICE guidelines. Data were collected in May 2022 using electronic as well as paper records. Emergency as well as elective surgeries were included. Procedures included anterior resections, Hartmann’s type operations, and abdomino-perineal resections (APERs). Patients who did not undergo surgery or underwent non-resectional surgery were excluded. Patients undergoing both elective and emergency operations were included. The data collection process involved the following: extracting demographic characteristics (age, gender), recording the type of procedure performed and the American Society of Anaesthesiologists (ASA) score, and collecting data on postoperative outcomes, including length of stay, complications, mortality, and return to theatre.

Data were analysed using Microsoft Excel (Microsoft Corp., Redmond, WA) and SPSS (IBM Corp., Armonk, NY). Means and standard deviation were calculated for quantitative variables such as age and length of stay, and frequencies were calculated for qualitative variables such as type of surgery, mode of surgery, mortality, and return to theatre. Results were compared nationally and internationally, including outcomes such as post-operative complications, 90-day mortality, return to theatre, length of stay, stoma formation, and stoma closure rate.

## Results

A total of 104 patients were included according to the inclusion criteria. The mean age was 69.13±9.45 years (median 70, range 45-98 years). Of the patients, 65 (62.5%) patients were male and 39 (37.5%) patients were female. Mean length of stay was 11.31±10.2 days (median 9, range 2-78 days). Laparoscopic surgery was performed in 51 (49%, N=104) patients, while open surgery was performed in 53 (51%, N=104) patients. Rate of complications was higher in the open group (30%, N=16), which also included the emergency patients, as compared to the laparoscopic group (17%, N=9).

Elective operations were performed in 96 (92.3%, N=104) patients, while emergency operations were performed in eight (7.7%, N=104) patients. Four patients in the emergency surgery group developed complications in the post-operative period, which included chest infection, ileus, anastomotic leak, and pulmonary embolism.

The rate of mortality in our study was 2.8% (N=3). Two of our patients who had emergency operations died within 30 days after the surgery, one due to severe chest infection and the second due to sepsis because of peritonitis, while one patient died within 90 days after developing severe sepsis secondary to a pelvic collection.

**Figure 1:**
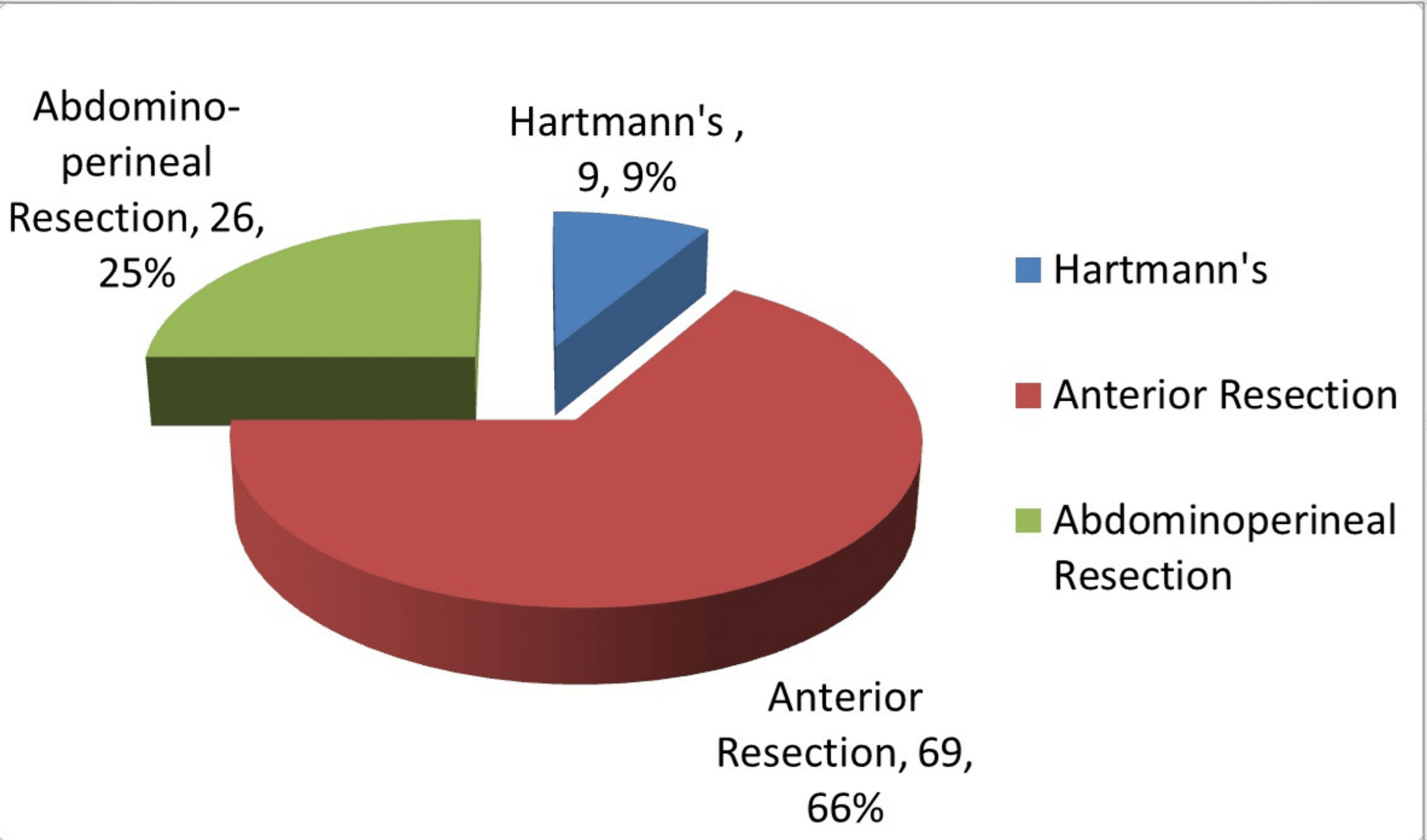
Breakdown of procedures performed (N=104)

**Figure 2:**
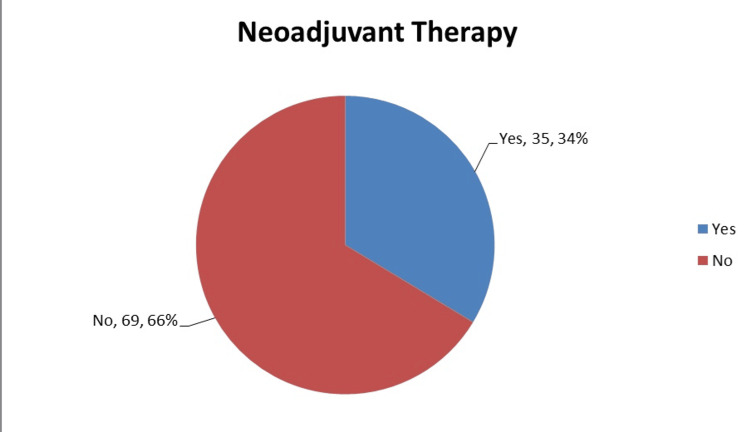
Patients receiving neoadjuvant therapy (N=104)

**Figure 3:**
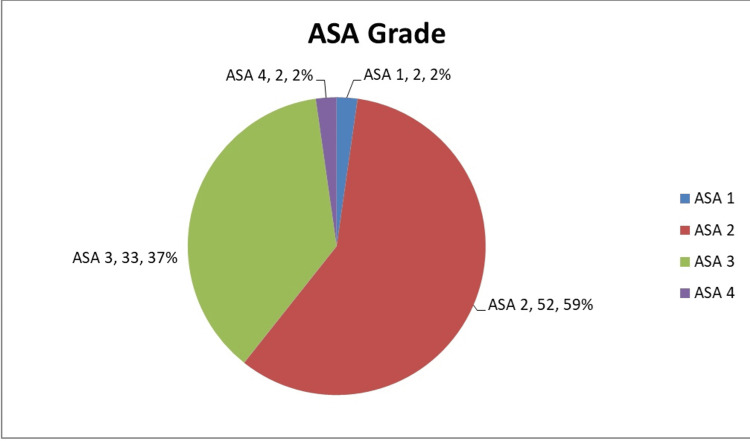
Distribution of ASA grade in our patients (N=104) ASA, American Society of Anesthesiologists

**Figure 4:**
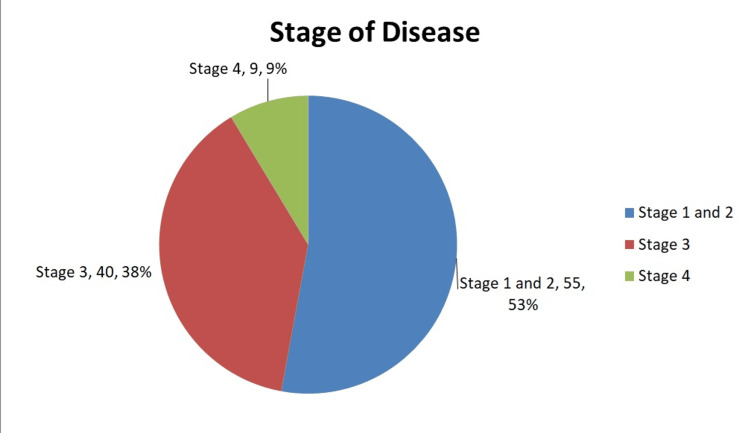
Division of stage of rectal cancer in our patients (N=104)

Anterior resection was the most commonly performed operation (66%, N=69), followed by APER (25%, N=26) and Hartmann’s type procedures (9%, N=9). Figure 1 illustrates the division of procedures performed. Nine (8.6%, n=9) patients had resectable hepatic metastases at the time of surgery. Figure 2 illustrates the patients who received neoadjuvant therapy. Seven (20%, n=35) of these patients developed post-operative complications, which was comparable to the rate of complications in the non-neoadjuvant group (19%, n=69). The complications are demonstrated in Table 1. Figures 3 demonstrates the distributions of ASA grade in our patients. Table 2 illustrates the tumour, node, and metastasis distribution in our patients, while Figure 4 demonstrates the stage of disease.

**Table 1:** Table detailing the frequency of various complications in our study (N=104) DVT, deep vein thrombosis; PE, pulmonary embolism

Ileus	5 (4.8)
DVT/PE	1 (1%)
Stroke	1 (1%)
Atrial fibrillation	2 (2%)
Acute kidney injury	4 (3.8%)
Anastomotic leak (managed by radiological drainage)	3 (2.8%)
Anastomotic leak (managed by re-operation)	2 (1.9%)
Pneumonia	1 (1%)
Intra-abdominal sepsis	6 (5.7%)
Total	25 (24%)

**Table 2:** Table demonstrating the Tumor, Node, Metastasis Distribution in our patients (n=104) Table demonstrating the tumour, node, and metastasis distribution in our patients (N=104)

Tumour, node, and metastasis distribution	
T0	3 (2.9%)
T1	11 (10.2%)
T2	21 (19.4%)
T3	50 (46.3%)
T4	19 (17.6%)
N0	64 (61.5%)
N1	21 (20.1%)
N2	19 (18.2%)
M0	95 (91.3%)
M1	9 (8.6%)

## Discussion

We reviewed the surgical management of rectal cancer in in our centre in this study. We operated on slightly older patients as compared to national and international studies. The mean age in our study was higher (69.13 years) as compared to 66.6 years in the group having laparoscopic surgery in the study by Tilney et al. [6]. Median age in our study was also higher, 70 years (range 45-98 years), as compared to 66 years (59-75 years) in Luvisetto et al.’s study and 65 years (37-89 years) in Petropoulou and Amin et al.’s study [8,13]. Comparing internationally, we operated on older patients as compared to Gilshtein et Al. (median 61 years, range 34-83), Liang et al. (73.5 % patients aged less than 71 years), and Molnar et al. (mean 66 ± 11.1 years) [14-16]. The gender distribution in our study was similar to that of national and international studies. The distribution of stage of disease in our patients was comparable to national and international studies, with majority of patients belonging to stages 1 and 2 followed by stage 3 [13,14,16].

The median length of stay in our study was nine (range 2-78) days, which is slightly higher than that of our peers, six days (2-30 days) in the study by Petropoulou and Amin, seven (5-13 days) days in the study by Luvisetto et al., and seven (5-11 days) days in the study by Tilney et al. Internationally Gilshtein et. al had a length of stay of 7 days (5-18 days) [6, 8, 13, 14]. Our median length of stay also included patients having APER, which tend to have a longer stay in hospital post-operatively. Another factor to take into consideration is the age of our patients, which is higher than our peers.

A total of five (4.8%) patients had anastomotic leak in our study, and these patients were diagnosed clinically and radiologically. Three (2.8%) of these patients were managed with a radiologically inserted drain, while two (1.9%) patients had to return to the theatre. Both the patients had their anastomosis taken down at re-operation and had stomas made. Six patients had intra-abdominal sepsis, which was not a result of a leak, and these patients were managed conservatively. Our leak rates were comparable nationally with our peers, who reported rates of 5.3%, 12.9%, and 2.4% [6,8,13]. Comparing internationally, our rate of leak, resulting in a return to theatre, was similar to those reported by international studies. Liang et al reported a leak rate of 3%, while Molnar and Gislhtein reported a leak rate of 3% and 1.8%, respectively [14-16].

Anterior resection was the most commonly performed surgery in our patients. Overall, 69 (66.3%) patients underwent anterior resections. Covering stomas were made for 52 patients, while 17 (25%, N=69) patients did not have any stoma formation. Covering stomas were reversed within 18 months in 24 (35%, N=69) patients. The rest of the patients did not have their stomas reversed within 18 months, due to various reasons including developing metastases, patient choice, and stricture formation.

The rate of recurrence at the time of data collection was 25% (N=26). Local recurrence occurred in four (3.8%) patients, while distant recurrence occurred in 20 (19.2%) patients. Local recurrence along with distant recurrence occurred in two (1.9%) patients. This rate of recurrence was also comparable to that reported by our national and international peers [13,17]. The number of patients who had positive circumferential resection margins was seven (6.7%). Despite this, our rate of recurrences remains comparable.

The rate of mortality in our patients was 2.8% (N=3). Two of these patients underwent emergency operations and died within 30 days, while one patient underwent elective surgery and died within 90 days. The rate of mortality in our study is slightly higher than those of our peers as we have included patients undergoing emergency operations as well as elective operations, as patients undergoing emergency operations are not physiologically optimised and tend to present in a compromised state.

The major limitation of our study is its retrospective nature and the fact that it is a single-centre study. Other areas of improvement include the low rate of laparoscopic surgery. The rate of laparoscopic surgery was lower as compared to open surgery in our centre, as one of our surgeons was not trained in laparoscopy and we also performed most of our APERs via open technique. Newer additions to our workforce have improved this rate in the last few years, and this will continue to improve over time.

Our study demonstrates that rectal cancer surgery can be safely performed in district hospitals with adequately trained surgeons and good basic facilities. The mean age of our patients was higher than that reported in other studies, which may have influenced outcomes such as length of stay and complication rates. Despite this, our mortality rate of 2.8% and anastomotic leak rate of 4.8% are comparable to those reported in the literature. The lower rate of laparoscopic surgery in our centre highlights the need for ongoing training and resource allocation to improve minimally invasive surgery rates.

Rectal cancer surgery continues to be performed in many district hospitals, and the push to centralisation continues. However, the impact of this on patients, including access and changes to waiting times, should be considered. Centralisation may impact the workforce as well and might result in de-skilling of the colorectal workforce, which may have an impact on patient care particularly at district hospitals. We postulate that district hospitals with well-trained workforce and access to intensive care and interventional radiology can treat rectal cancer using a multi-disciplinary approach.

## Conclusions

Rectal cancer continues to be treated in many district hospitals across the world, and the recent push for centralisation and hyper-specialisation of rectal surgery might hold merit but we demonstrated that rectal cancer surgery can be safely undertaken in district hospitals with good results using a combination of laparoscopic and open surgery. This requires adequately trained colorectal surgeons and access to good support infrastructure including intensive care, interventional radiology, oncology, and cancer nurse specialists. Future research should focus on prospective, multi-centre studies to validate these findings. Additionally, healthcare policy should consider the potential benefits of maintaining rectal cancer surgery capabilities in district hospitals to ensure patient access to care and support the training of colorectal surgeons.
